# Envenomization with Snake Versus Scorpion Venom: Postmortem Repercussions on the Successional Dynamics of Carcass-Attracted Flies in a Forensic Context

**DOI:** 10.3390/insects17080863

**Published:** 2026-08-19

**Authors:** Afnan Saleh Al-Qurashi, Mohammed Saleh Al-Khalifa, Hathal Mohammed Al Dhafer, Mahmoud Saleh Abdel-Dayem, Hossam Ebaid, Ashraf Mohamed Ahmed

**Affiliations:** 1 Zoology Department, College of Science, King Saud University, Riyadh 11451, Saudi Arabia; 443203299@student.ksu.edu.sa (A.S.A.-Q.); mkhalifa@ksu.edu.sa (M.S.A.-K.); habdrabou@ksu.edu.sa (H.E.); 2 Plant Protection Department, College of Food and Agriculture Sciences, King Saud University, Riyadh 11451, Saudi Arabia; hdhafer@ksu.edu.sa (H.M.A.D.); mseleem@ksu.edu.sa (M.S.A.-D.)

**Keywords:** Diptera, forensic, flies, carcass, snake, scorpion, venoms, envenomization

## Abstract

Background: Animal venoms may alter postmortem carcass conditions and thereby influence carrion-associated insects. However, it remains unclear whether lethal envenomization affects adult Diptera as a broad assemblage-level response or as taxon-specific changes during decomposition. Methods: Fifteen rabbits were assigned to control, snake-envenomized, or scorpion-envenomized treatments (with five carcasses each). Carcasses were exposed under semi-natural conditions, until complete dryness, over 11 days postmortem. Adult flies were collected daily for analyzing abundance at total, family, and selected species levels, using negative GLMM, with carcass identity as a random effect and postmortem day as a nonlinear predictor. Results: A total of 5497 adult flies representing 13 species in seven families was recorded. Total fly abundance was similar among treatments and was mainly structured by postmortem day. In contrast, treatment-related temporal patterns were detected at finer taxonomic levels, especially at the family level for Muscidae, Ephydridae, and Sarcophagidae; and at the species level for the sarcophagid *Sarcophaga argyrostoma* and the ephydrid *Actocetor indicus* only. Conclusions: both snake and scorpion envenomization did not produce a broad shift in total adult-fly abundance, but in selected Dipteran taxa showing treatment-related responses during particular postmortem periods. These findings should be interpreted cautiously as exploratory observations rather than evidence of a robust venom effect on adult fly communities.

## Simple Summary

This study examined whether snake or scorpion envenomization influences the abundance and daily occurrence of adult flies on decomposing rabbit carcasses. The main question was not only whether envenomized carcasses attract more or fewer flies, but also whether any effect appears across the whole fly assemblage or only in particular taxa. Total fly abundance was primarily driven by postmortem day and did not differ significantly among treatments. However, treatment-related differences were detected in specific fly families (Muscidae, Ephydridae, and Sarcophagidae) and species (the arcophagid *Sarcophaga argyrostoma* and the ephydrid *Actocetor indicus*). These findings indicate that venom-related effects were expressed mainly at family and species levels rather than in overall fly abundance, highlighting the importance of taxonomic identification of flies to family and species levels when interpreting insect evidence from suspected envenomation cases.

## 1. Introduction

Forensic entomology is a field of study that involves using carcass-attracted necrophagous insects to aid in criminal investigations. These insects have the capability to arrive at a carcass within minutes postmortem to form distinct carcass-associated communities [1,2]. Understanding their successional pattern, behavior, taxonomy, and physiology can aid in providing evidence for unraveling suspicious crimes [1,3] and, hence, establishing important forensic evidence [1,4,5], such as providing physical evidence of the timing, location, and cause of death at the crime scene and, subsequently, aiding in estimating the postmortem interval (PMI) [1,2,6,7,8,9]. Colonization of carcasses by necrophagous insects varies depending on several factors: (a) the environmental conditions [10,11]; (b) the environmental site [12,13,14]; (c) the chemical components of the carcass body [15,16]; (d) the activity of the carcass-decomposing microorganisms [17,18]; (e) the accessibility to the carcass [19,20,21,22]; and (f) the presence of toxins in the carcass’s tissues [23,24,25,26]. 

While classical entomotoxicology has predominantly focused on the effects of xenobiotics on larval development, developmental rates, and subsequent PMI estimation [27,28,29,30,31,32,33], a separate but equally important question concerns whether toxic substances, including venoms, alter the attraction and succession patterns of adult necrophagous flies, a context that has both biological and forensic significance. Adult flies are the primary colonizers of carcasses, and their arrival timing and abundance determine the initial colonization events that set the stage for subsequent larval development [1,2,6]. Unlike larval stages, which are exposed to toxins primarily through tissue consumption, adult flies respond to volatile organic compounds (VOCs) and other olfactory cues emanating from the carcass [15,34,35]. Therefore, we hypothesize that envenomation alters carcass chemistry via changes in tissue decomposition pathways, microbial succession, or VOC profiles, which could influence adult fly attraction and visitation patterns independently of any direct toxicological effects on developing larvae. No published studies have directly demonstrated that snake or scorpion envenomation alters the VOC profiles, microbial communities, or decomposition chemistry of carcasses in ways that affect insect attraction.

Unlike drug and poison administration or environmental toxin exposure, envenomation with venomous animals involves complex mixtures of bioactive compounds that can persist in tissues and affect decomposition processes and colonization with associated insects [36,37,38,39,40]. Because bacterial communities regulate carrion VOC profiles, alterations in decomposition processes (as these VOCs serve as key olfactory cues for adult necrophagous flies) may indirectly influence fly colonization patterns [41]. Previous studies have demonstrated that snake and scorpion venoms can directly affect necrophagous insects by suppressing neuromuscular activity and altering larval development, morphology, and insect succession, providing a biological basis for venom-induced changes in forensic entomological evidence [28,39,42]. These studies illustrated that venoms can affect insects in general, but we emphasize that the pathway from venom exposure to altered adult attraction remains untested.

Globally, envenomation-related deaths constitute a significant health problem, especially in rural and underdeveloped areas in tropical and subtropical countries [43,44,45]. The majority of reported deaths are caused by arthropods (including scorpions) and snakes [43,46,47,48]. Venomous scorpions and snakes, in particular, are distributed throughout the globe [49,50,51,52,53,54,55,56,57,58,59,60,61]. Recent estimations showed that about 5.4 million people are bitten by snakes; of them, 1.8 to 2.7 million result in clinical illness, 81,410 to 137,880 deaths, and approximately 400,000 survivors suffering permanent disabilities annually [59,62,63]. In addition, approximately 1.2 million scorpion stings occur each year, leading to about 3250 deaths [64,65]. 

Deadly snakebite occurs predominantly in rural and remote settings, places where delayed access to medical treatment may increase the need for medico-legal investigation. Several autopsy-based studies and forensic case reports revealed that fatal snakebite deaths undergo forensic examination to establish the cause and manner of death and to exclude criminal involvement [66,67,68,69]. In India, a fatal cobra bite was reported in a case suspected to involve criminal activity [70]. In Brazil, nearly 200,000 stings and 133 deaths were recorded in 2023, with an estimated more than 2 million new cases of scorpion stings between 2025 and 2033 [71]. In Egypt, three children were killed as a result of snake bites, where snake envenomation is uncommon [72]. In the Middle East and Africa, thousands of snakebites, as well as scorpion and spider stings, were documented [50,65,72,73,74]. In addition, venom abuse and suicide are rapidly emerging phenomena in a forensic context. In this regard, [40] directed the attention towards venom abuse and presented some reported cases of venom consumption and addiction. Many venom-related cases of suicide have been reported for a long time [75,76,77,78,79]. Those cases suggest that venom abuse and addiction under their influence may constitute a threat to society because of the associated mortality and morbidity.

Saudi Arabia is one of the countries with high rates of snakebites [52,73,80,81,82,83,84] and scorpion stings [52,55,85,86,87,88]. Scorpions are distributed throughout the country regions, causing a significant incidence of approximately 14,500 stings recorded annually [87]. An overall 1328 cases were reported between 2015 and 2019 within the Saudi community; most of them were stings by arthropods and bites by snakes [80], and over 16,000 cases were hospitalized, with 15 fatalities reported [88]. However, few studies from Saudi Arabia have addressed envenomation from a forensic entomology perspective. Earlier work on lethal snake and scorpion envenomization examined postmortem decomposition and beetle succession [36]. Nevertheless, those findings cannot answer whether adult Diptera respond to envenomization as a pooled assemblage or through selected families and species. This is a separate forensic question because adult flies are often among the earliest and most abundant colonizers of carcasses [2], and their response to envenomization, whether assemblage-wide or taxon-specific, has direct implications for how forensic entomologists should interpret insect evidence in suspected envenomation cases. If the response is assemblage-wide, then simple measures of total fly abundance might suffice; if it is taxon-specific, then species-level identification becomes critical. The present study, therefore, tested whether lethal envenomization by the local snake *Walterinnesia aegyptia* or the fat-tail scorpion *Androctonus crassicauda* produces a broad shift in total adult fly abundance or whether any response is restricted to particular Diptera families and/or species during specific postmortem periods. Our a priori prediction was that postmortem day would remain the main driver of adult fly abundance. We also hypothesized that if venom-related effects exist, they would appear as taxon-specific and time-dependent changes rather than as a uniform change in the pooled fly catch. However, we acknowledge that this taxon-specific hypothesis was partly developed after observing initial patterns in the data. We therefore present the family- and species-level analyses as both confirmatory tests of the a priori prediction and exploratory examinations of finer-scale patterns. The taxon-specific findings should be interpreted as hypothesis-generating rather than as confirmatory evidence.

## 2. Materials and Methods

### 2.1. Study Period and Weather Parameters

This study was carried out over an 11-day period during the summer (6–17 June 2023). The atmospheric data were obtained from the Saudi National Center for Meteorology [89]. The atmospheric data obtained from the Saudi National Center for Meteorology were retrieved as daily meteorological records (daily minimum, maximum, and mean values), whereas on-site weather parameters were measured independently at the experimental site once daily at midday throughout the experimental period. The on-site weather parameters were recorded manually once each day at midday to characterize site conditions during the experiment; however, this approach was intended to provide a standardized daily snapshot rather than continuous microclimatic monitoring. The ambient temperature and relative humidity were measured using a digital thermometer and a hygrometer, respectively (Elitch Technology, Nanjing, China), following their manufacturer’s instructions. Wind speed was monitored using the Skywatch Wind Meter device (Skywatch^®^, Yver-don-les-Bains, Marin-Epagnier, Switzerland) following its manufacturer’s instructions. The meteorological dataset is represented in Table 1.

### 2.2. Experimental Site

This study was conducted in the botanical garden of the Botany and Microbiology Department, College of Science, King Saud University, Riyadh, Saudi Arabia (24°43.174′ N, 46°36.954′ E) (Figure S1A). The garden covers approximately 10,000 m^2^, has clayey sandy soil, and supports diverse grasses and herbs. Additionally, it encompasses a wide range of plant species, including endangered, wild, and medically and economically important plant species [90]. This garden was targeted because it offers a standardized, semi-natural, and plant-rich environment that matches ecological features found in field-based forensic scenarios where the forensic investigations are likely to take place.

### 2.3. Experimental Animals

#### 2.3.1. Rabbits

Although domestic pigs are commonly used as human analogs in forensic experiments [91], their use may be constrained by ethical, cultural, and logistical considerations in some regions [92], including Saudi Arabia. Rabbits have nevertheless been widely used as experimental models in forensic entomology studies, including studies conducted under Saudi environmental conditions [14,36,93,94,95]. Thus, males of *Oryctolagus cuniculus domesticus* (Linnaeus, 1758) domestic rabbits were used as experimental models for conducting the experiments in this study (Figure S1B) according to [96]. Rabbits (≈3.0 kg each), with minimal variation in body weight, were purchased at a local market in Riyadh City, Saudi Arabia. Prior to the experiments, animals were first housed for acclimatization for 3 days under standardized rearing conditions in the Animal House, Zoology Department, College of Science, King Saud University, with food and water *ad libitum*, before use in the experiments.

#### 2.3.2. Venomous Animals

Two local venomous animals were targeted as different sources of neurotoxic venoms: the black snake, *Walterinnesia aegyptia* Lataste, 1887 (Squamata, Elapidae) [52,97,98,99] (Figure S1C; from [100]) and the fat-tail scorpion, *Androctonus crassicauda* (Olivier, 1807) (Scorpiones, Buthidae) [55,101,102,103] (Figure S1D; from [104]). Both animals are being maintained in the Animal House, Zoology Department, College of Science, King Saud University, under standard rearing conditions according to [105] and [106], respectively. Dealing with snakes and scorpions was carried out by a qualified specialist from the Herpetology Laboratory in the Zoology Department.

### 2.4. Venom Preparation and Lethality Assay

Venoms were freshly collected and prepared from scorpions and snakes as detailed in [36]. Prior to each experiment, each venom aliquot was freshly prepared in 0.9% saline (pH 7.2), according to [107] in a final concentration of 1.0 mg/mL (*w*/*v*). To determine the LD_50_ and LD_95_ values of each venom, a lethality bioassay was conducted using Swiss albino mice according to the World Health Organization guidelines [108] as detailed in [36]. Probit analysis revealed that the experimentally determined LD_50_ and LD_95_ values of *W. aegyptia* venom were 0.053 and 0.066 mg/kg, respectively, whereas those of *A. crassicauda* venom were 1.416 and 2.516 mg/kg, respectively [36]. Handling snakes and scorpions, and performing venom collection, were carried out by a qualified specialist from the Herpetology Laboratory in the Zoology Department.

### 2.5. Rabbits’ Envenomization 

The equivalent dose of LD_95_ for rabbits was calculated for each venom, for use in killing the experimental rabbits, according to [109] and as detailed in [36]. Then, each animal was intravenously injected in the ear vein with a freshly prepared 500 µL venom aliquot containing 0.264 mg or 10.064 mg/rabbit of the *W. aegyptia* snake or *A. crassicauda* scorpion, respectively, based on a mean rabbit body mass of about 3.0 kg, according to [24] (Figure S1E). Rabbits weighed between 2.5 and 3.0 kg. Because body weight variation was limited, a uniform fixed dose was used to ensure rapid and standardized lethal envenomization and a consistent onset of the postmortem interval among carcasses following [36,37].

### 2.6. Experimental Design

**Figure 1:**
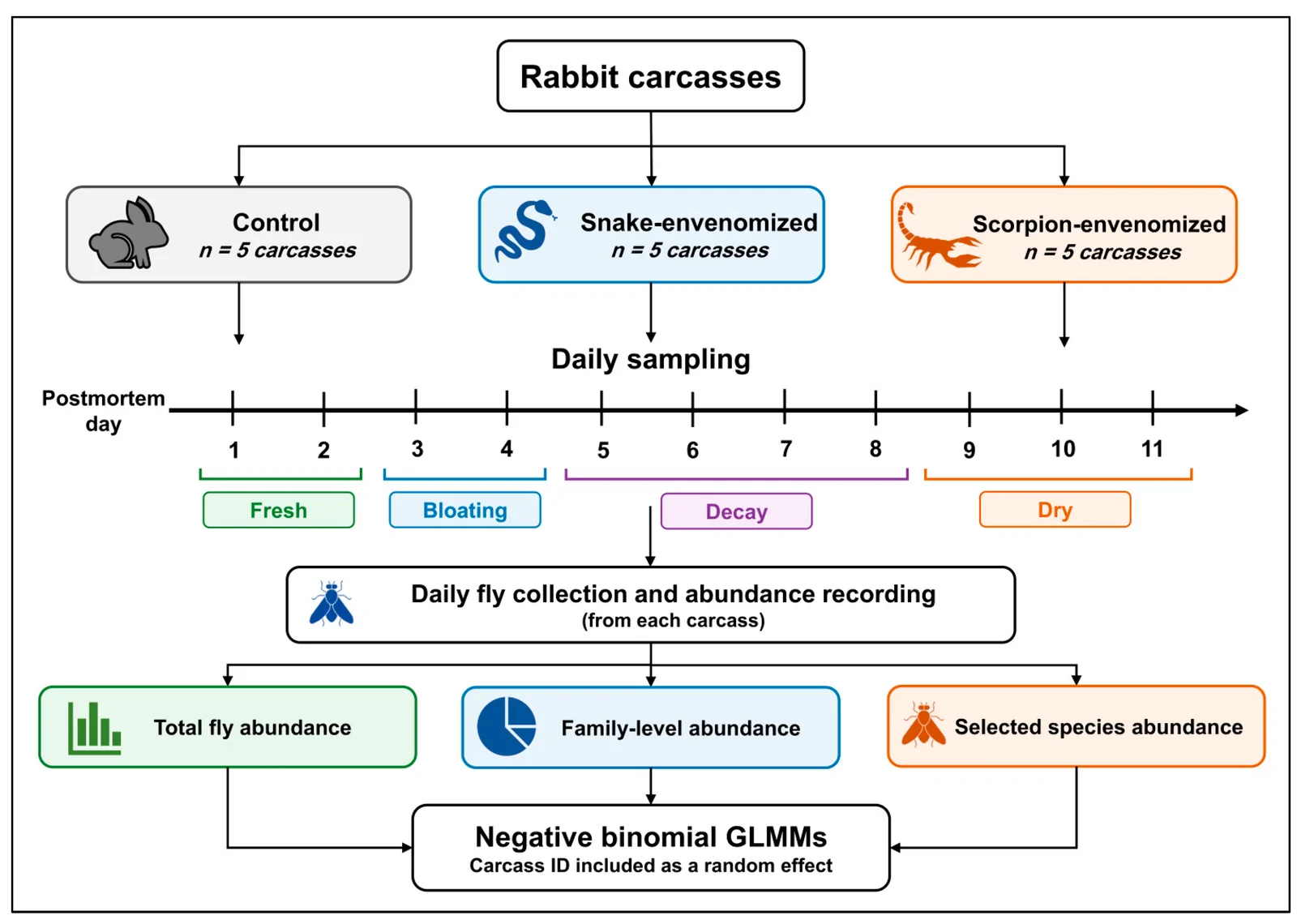
A schematic diagram summarizes the experimental design, insect sampling timeline, and analytical workflow for fly-abundance succession pattern assessments over the experimental period.

The experimental design, including the daily sampling timeline, decomposition stages, and analytical workflow, is summarized in Figure 1. Briefly, a total of fifteen rabbits were divided into three groups (5 animals each; *n* = 5). Rabbits in the first and second groups were intravenously envenomized with 0.264 mg/rabbit of the *W. aegyptia* snake venom and 10.064 mg/rabbit of *A. crassicauda* scorpion venom, respectively. The death of envenomized animals was confirmed within 20 min post-injection. In parallel, rabbits in the third group (control) received 0.9% saline and were then euthanized with CO_2_, according to [110]. Rabbit carcasses were immediately translocated to the experimental site within 30 min post-death and placed individually in a protective metal cage, as detailed in [95]. Carcasses were spaced at 10 meters apart to provide isolated resources for carcass-seeking insects, as recommended by [111], in order to minimize the interference of odors on insect attraction [112]. Pitfall traps were placed adjacent to each carcass throughout the experimental period to continuously sample insects between the scheduled collection periods, as recommended by [113] and following [14]. All experimental carcasses were placed in standardized shady places underneath trees (Figure S1B) in order to avoid the direct sunlight, which is known to impact insect succession [114]. Dealing with rabbits, snakes, and scorpions; injecting and killing rabbits; insect collection; and conducting experiments were approved by the Research Ethics Committee at King Saud University (approval code: KSU-SE-23-83).

### 2.7. Fly Collection and Identification

Carcass-associated flies were collected twice a day, at 9.00 a.m. and 5.00 p.m., immediately after the experiment was set up until complete dryness of the carcasses, according to [115]. Adult flies were collected using a standard entomological sweep net and pitfall traps (five repeated nets/carcass at each visit), as recommended by [116] and following [14]. Live adult flies were killed at −20 °C, then preserved in 70% ethanol as detailed in [14]. Flies were counted and morphologically identified to the species level at the King Saud University Museum of Arthropods (KSMA), using established taxonomic keys for Diptera [117,118], where voucher materials of the study have been deposited.

### 2.8. Statistical Analysis

Statistical analyses were conducted in R version 4.5.2 (R Core Team, 2025). Fly abundance data were analyzed using negative binomial generalized linear mixed models (GLMM) fitted with the glmmTMB package [119]. Carcass identity was included as a random effect to account for repeated observations from the same carcass. Postmortem day was modeled as a nonlinear continuous predictor using natural cubic splines. Treatment, nonlinear postmortem day, and their interaction were evaluated as fixed effects; when the interaction was not supported, simpler additive models were used for interpretation. Model adequacy was assessed using simulation-based residual diagnostics implemented in the DHARMa package [120]. Fixed effects model terms were evaluated using likelihood-ratio tests. Estimated marginal means and Tukey-adjusted pairwise contrasts were calculated on the response scale using the emmeans package [121]. Model-predicted means and observed daily means ± SE were visualized using the ggplot2 package [122]. Families or species with very sparse counts were summarized descriptively rather than included in abundance-based GLMM interpretation. When only one individual of a species is reported on a carcass, it is considered a singleton; two individuals are considered a doubleton, according to [123], and following [14,36,39,95,114].

## 3. Results

### 3.1. Weather Conditions

Weather conditions remained generally stable throughout the 11-day experimental period (Table 1). According to the Saudi National Center for Meteorology, the mean maximum and minimum temperatures were 41.0 °C and 29.27 °C, respectively, with an overall mean of 35.14 °C. Relative humidity (RH) remained low, ranging from 5.0% to 13.0% with a mean of 7.8%. Wind temperature remained relatively stable and averaged 32.03 °C, whereas wind speed ranged from 5 to 31 km/h, with a mean of 13.15 km/h. These values fall within the normal range for this period in the Riyadh region. The manually recorded on-site data showed a similar pattern (Table 1). Ambient temperature ranged from 29.0 to 38.0 °C, with a mean of 34.0 °C, whereas RH ranged from 30.0 to 36.0%, with an average of 32.0%. Wind temperature varied from 29.0 to 34.0 °C, with a mean of 31.37 °C. A wide variation was noticeable in wind speed, as it ranged from 0.0 to 18.0 km/h but remained low overall, with a mean of 3.25 km/h.

### 3.2. Total Fly Abundance Pattern

**Figure 2:**
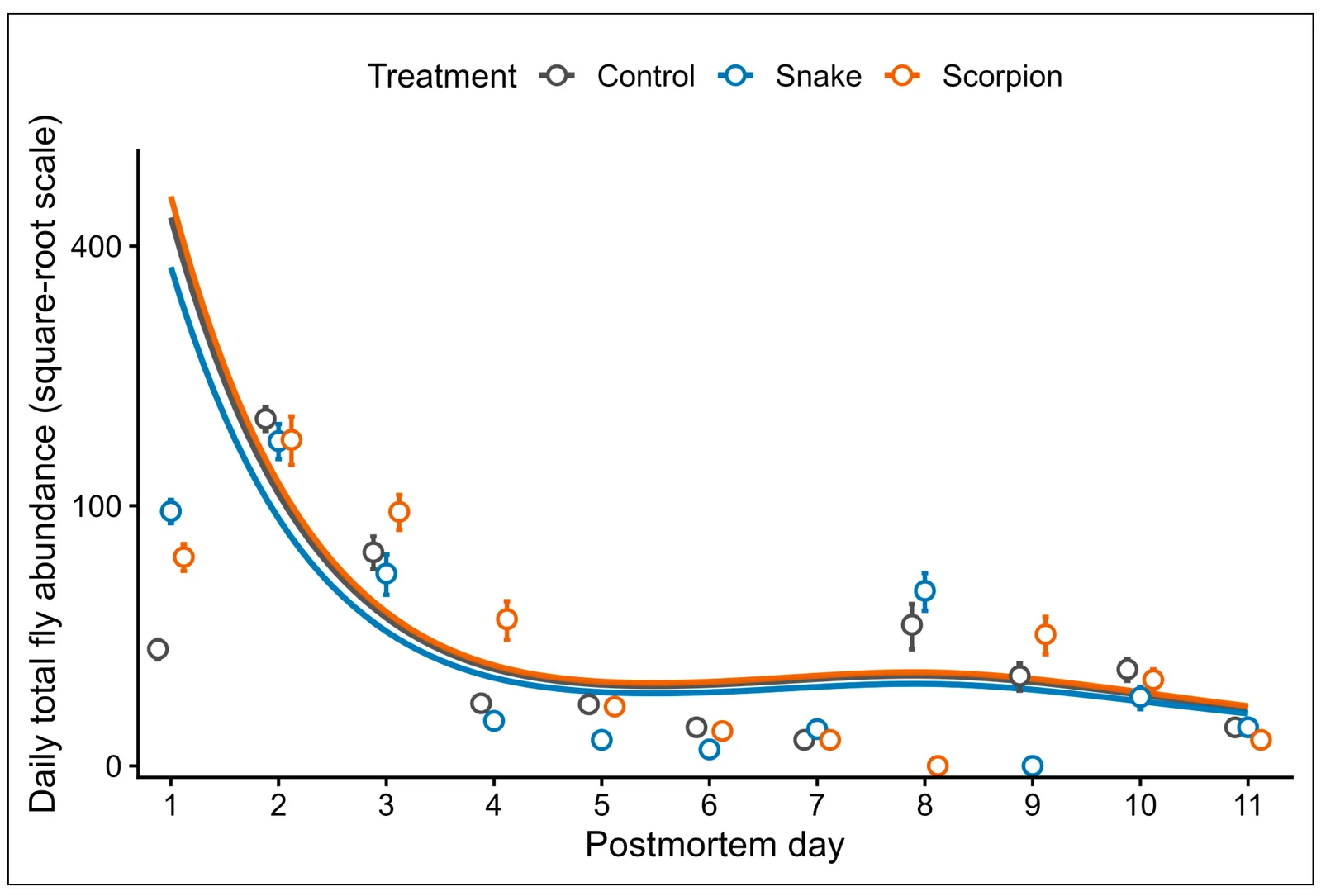
Daily total fly abundance across postmortem days in control, snake-envenomized, and scorpion-envenomized carcasses. Lines represent model-predicted means from the selected negative binomial generalized linear mixed model. Points and error bars represent observed daily means ± SE and are slightly offset horizontally for visibility. The y-axis is shown on a square-root scale to improve visualization of early high-abundance values.

A total of 5497 adult flies were collected from all experimental carcasses over the 11-day experimental period. The total catch was distributed almost equally among treatments, with 1839 individuals (33.45%) recovered from snake-envenomized carcasses, 1846 individuals (33.58%) from scorpion-envenomized carcasses, and 1812 (32.96%) from control carcasses. Daily total fly abundance varied mainly with postmortem time rather than carcass treatment. The interaction between treatment and nonlinear postmortem day was not significant, although it approached the 0.05 threshold (LRT χ^2^ = 11.32, df = 6, *p* = 0.079; Table 2). Therefore, the simpler additive GLMM was used for interpretation. In this model, postmortem day had a strong effect on total fly abundance (LRT χ^2^ = 103.10, df = 3, *p* < 0.001), whereas treatment did not significantly improve model fit after accounting for nonlinear temporal change and repeated observations within carcasses (LRT χ^2^ = 1.26, df = 2, *p* = 0.533; Table 2). Model-predicted and observed daily means showed a clear temporal pattern (Figure 2). Total fly abundance was highest during the early postmortem period, declined sharply over subsequent days, and remained low during the later decomposition period. Treatment-specific curves showed no clear separation, consistent with the non-significant treatment effect in the selected model.

### 3.3. Family-Level Abundance Pattern

Seven dipteran families were recorded during the study (Table S1). Muscidae and Calliphoridae had the highest total abundances (51.17 and 43.46%, respectively), followed by Sarcophagidae and Ephydridae (2.45 and 2.30%, respectively), whereas Phoridae, Ulidiidae, and Drosophilidae were recorded in low numbers (0.34, 0.21, and 0.05%, respectively). 

**Figure 3:**
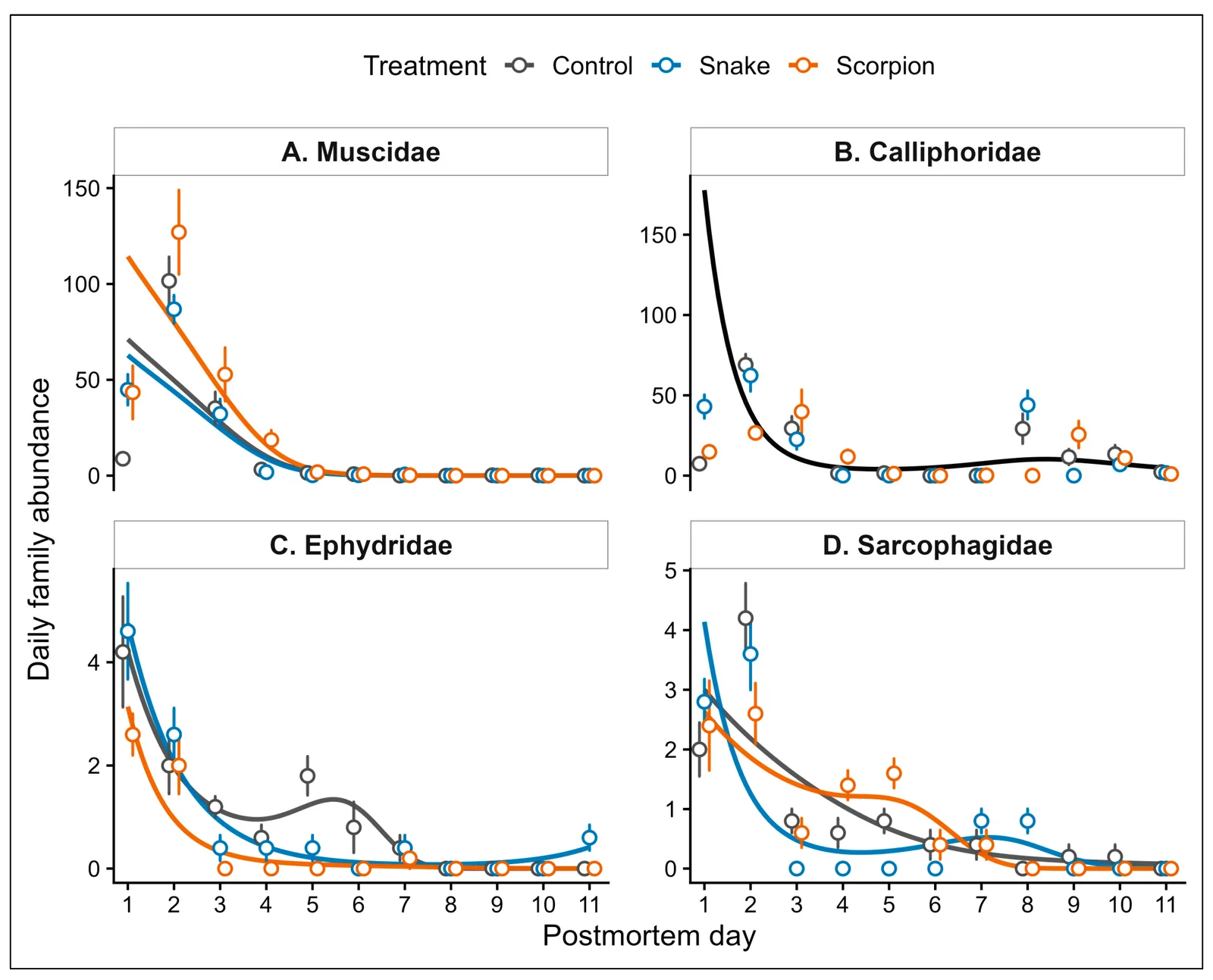
Predicted abundance patterns of the main dipteran families across postmortem days. Lines represent model-predicted means from the selected negative binomial generalized linear mixed models for Muscidae, Calliphoridae, Ephydridae, and Sarcophagidae. For Calliphoridae, the selected model included postmortem day only; therefore, a single overall predicted curve is shown. Points and error bars represent observed daily means ± SE and are slightly offset horizontally for visibility. Note that y-axis scales differ among panels to improve visualization of families with lower abundance.

For Muscidae, the treatment-nonlinear postmortem-day interaction was not supported, and the additive model was used for interpretation (Table 2). In the selected model, both treatment and nonlinear postmortem day affected Muscidae abundance (Table 2). Tukey-adjusted contrasts showed higher Muscidae abundance in scorpion-envenomized carcasses than in snake-envenomized ones, whereas the control treatment did not differ from either envenomized treatment (Table 3). The predicted abundance curves showed a marked early peak followed by a rapid decline through the later postmortem period (Figure 3A).

Calliphoridae showed a different pattern. Its abundance changed significantly with postmortem day, but not by treatment (Table 2). Consistent with the selected day-only model, the predicted calliphorid curve declined sharply across the postmortem period, while the observed treatment means showed no clear separation among carcass treatments (Figure 3B).

Among the less abundant families, Ephydridae showed significant treatment-related temporal variation (Table 2). The strongest contrasts occurred during the middle part of decomposition. Predicted Ephydridae abundance was higher in control carcasses than in scorpion-envenomized carcasses on days 4–6 and higher in control than snake-envenomized carcasses on days 5–6 (Figure 3C; Table 3). Sarcophagidae also showed significant temporal and treatment-related variation (Table 2). Tukey-adjusted contrasts showed lower predicted abundance in snake-envenomized carcasses than in control or scorpion-envenomized carcasses during days 3–5 (Figure 3D; Table 3).

Phoridae, Ulidiidae, and Drosophilidae had low total abundances and high proportions of zero observations. These families were therefore summarized descriptively and were not included in abundance-based GLMM interpretation (Table S1).

### 3.4. Species-Level Differential Succession

Thirteen dipteran fly species were recorded during the study (Table 4). Of them, *Chrysomya albiceps* (Wiedemann, 1819) (Calliphoridae), *Musca sorbens* Wiedemann, 1830 and *Musca domestica* Linnaeus, 1758 (Muscidae), *Sarcophaga argyrostoma* (Robineau-Desvoidy, 1830) (Sarcophagidae), and *Actocetor indicus* (Schiner, 1868) (Ephydridae) were the most abundant species. These species were included in the species-level GLMM analyses. The remaining species were summarized descriptively because of their low total abundance and high proportions of zero observations.

**Figure 4:**
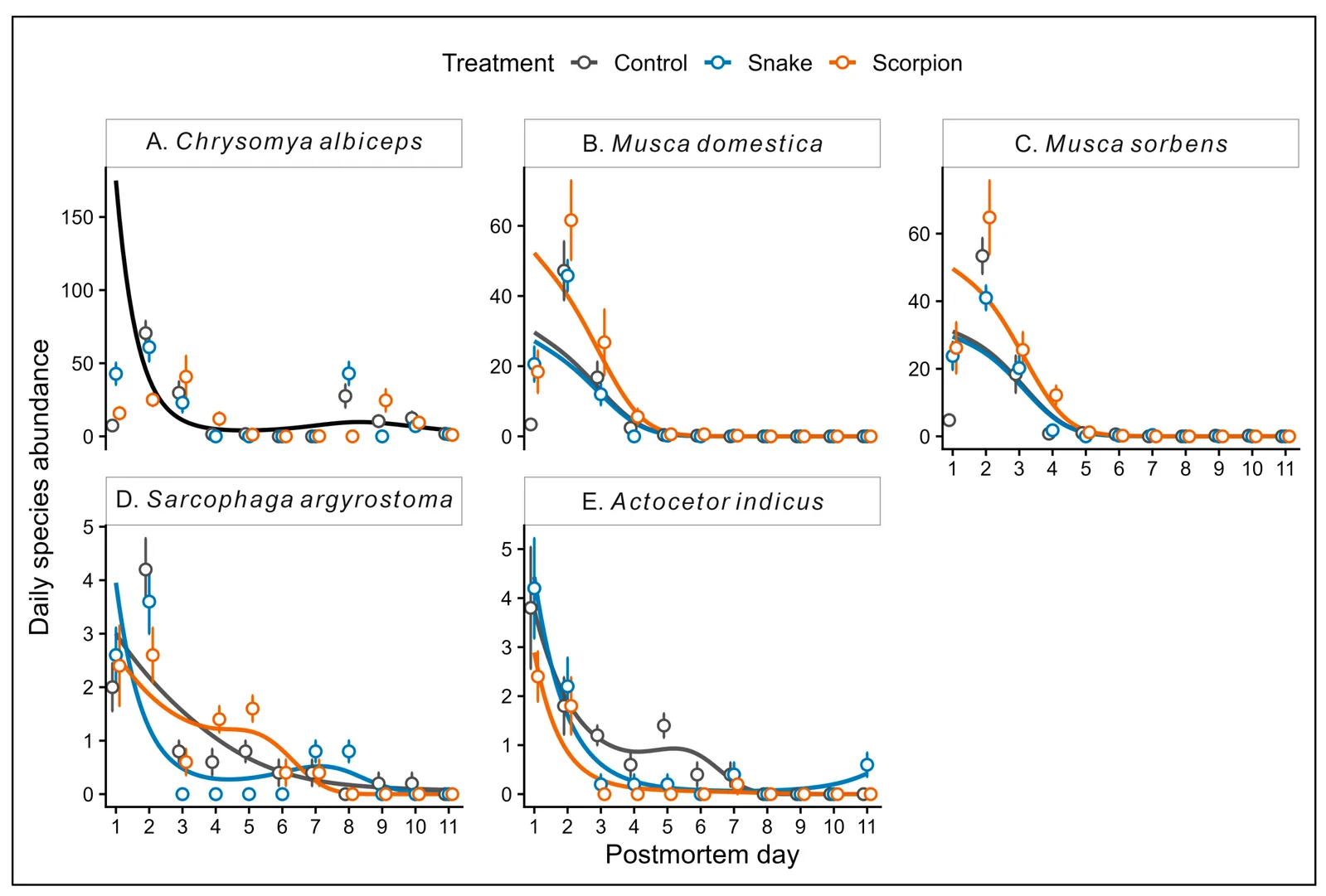
Predicted abundance patterns of selected fly species across postmortem days. Lines represent model-predicted means from the selected negative binomial GLMM for *C. albiceps*, *M. domestica*, *M. sorbens*, *S. argyrostoma*, and *A. indicus*. For *C. albiceps*, the selected model included postmortem day only; therefore, a single overall predicted curve is shown. Error bars represent observed daily means ± SE and are slightly offset horizontally for visibility.

Species-level analyses showed that the dominant species were primarily structured by postmortem time, although treatment-related effects were detected in some taxa (Figure 4; Table 5). Here, dominant species refers to the species that consistently constituted the core adult-fly assemblage throughout the experiment. They were selected because they consistently occurred throughout the experiment and provided sufficient observations for abundance-based GLMM analyses. Rare species were retained descriptively and were not included in the abundance-based GLMM analyses because of insufficient observations.

The dominant calliphorid species, *Chrysomya albiceps*, was strongly affected by nonlinear postmortem day, but neither treatment nor the treatment-day interaction significantly improved model fit (Table 5). Its predicted daily abundance followed a clear temporal successional pattern, with no evident separation among treatments in the observed means (Figure 4A).

Among Muscidae, both *Musca domestica* and *Musca sorbens* showed strong nonlinear temporal patterns (Figure 4B,C; Table 5). *Musca domestica* also showed a significant overall treatment effect, although Tukey-adjusted pairwise comparisons did not clearly separate individual treatments. *Musca sorbens* showed only a marginal treatment effect, with scorpion-envenomized carcasses tending to support higher predicted abundance than snake-envenomized carcasses, but this contrast did not remain significant after Tukey adjustment.

*Sarcophaga argyrostoma* showed the clearest treatment-specific temporal response among the modeled species. The treatment × nonlinear day interaction was significant (Table 4). Tukey-adjusted contrasts showed lower predicted abundance in snake-envenomized carcasses than in control on days 3 and 4 (Table 6). Predicted abundance was also lower in snake-envenomized carcasses than in scorpion-envenomized carcasses during the mid-successional period, especially on days 3–5 (Figure 4D; Table 6). 

The results for *Actocetor indicus* showed treatment-related and temporal variation, but these should be interpreted cautiously because of lower abundance and a high proportion of zero observations (Table 5). Tukey-adjusted contrasts showed higher predicted abundance in control carcasses than in scorpion-envenomized carcasses on days 4 and 5 and higher predicted abundance in control carcasses than in snake-envenomized carcasses on days 4–6 (Figure 4E; Table 6).

The remaining rare species, including *Atherigona orientalis* Schiner, 1868 (Diptera: Muscidae), *Chrysomya marginalis* (Wiedemann, 1830) (Diptera: Calliphoridae), *Blaesoxipha rufipes* (Macquart, 1843) (Diptera: Sarcophagidae)*, Brachydeutera longipes* Hendel, 1913 (Diptera: Ephydridae), *Discomyza incurva* Fallén, 1823 (Diptera: Ephydridae), *Megaselia scalaris* (Loew, 1866) (Diptera: Phoridae), *Physiphora smaragdina* (Loew, 1844) (Diptera: Ulidiidae), and *Leucophenga* sp. (Diptera: Drosophilidae) were not included in abundance-based GLMM interpretation because of their sparse counts (Table 4).

## 4. Discussion

Envenomation-related death cases in the forensic context are highlighted by the substantial global and regional incidence of venomous animal bites and stings. In this regard, severe morbidity and thousands of deaths are being reported each year worldwide from snakebites and scorpion stings [61,124]. These facts underline the need for understanding how lethal envenomization may influence postmortem decomposition of the cadaver and the succession of associated insects in the forensic context. Addressing this knowledge gap with more studies may, on one hand, contribute to the enhancement of the PMI estimation accuracy and, on the other hand, provide insight into the differentiation between causes of death involving venomous animals. This, consequently, will aid in the advancement of forensic science in regions such as Egypt and Saudi Arabia, as they are among the countries with high rates of snakebites [52,73,80,81,82,83,84,125] and scorpion stings [52,55,85,86,87,88,108,126,127]. To the best of our knowledge, no such studies have been conducted in both countries except [28] in Egypt. Hence, a long-term project was started for addressing this knowledge gap in Egypt [37,38,39] and Saudi Arabia [36]. 

The present study extended our research to comparatively investigate fly succession on snake- and scorpion-envenomized carcasses under comparable environmental conditions. This is to address a distinct forensic question from previous work on envenomized carcasses. Our previous study on the same venom treatments focused on decomposition and beetle succession [36]. In contrast, the present analysis tested whether adult Diptera respond to envenomization at the pooled assemblage level or only through selected families and species. This distinction is important because flies and beetles differ in their arrival time, resource use, decomposition-stage association, and forensic interpretation [6,13,14,24,25,36,128,129]. The present study shows that venom-related effects were not expressed as a broad change in the pooled fly catch, but as taxon-specific and time-dependent patterns. However, these patterns should be interpreted as observations from a single summer experiment at one locality, using rabbit carcasses, rather than as evidence of a generalizable effect of envenomization on adult Diptera. The treatment signal was concentrated in particular taxa and at particular postmortem days, and the affected taxa were substantially less abundant than the dominant ones. These observations require independent replication before they can be interpreted as biologically meaningful venom effects.

### 4.1. Total Abundance Pattern and Taxon-Specific Response

Data from the present study revealed that postmortem day was the main factor structuring total fly abundance. After accounting for nonlinear temporal change and repeated observations from the same carcasses, snake and scorpion envenomization did not significantly alter total fly abundance. In this experiment, the pooled fly catch tracked decomposition time more closely than venom treatment. This pattern is consistent with the general role of Diptera as early and abundant colonizers of vertebrate remains, especially during the early stages of decomposition [1,2], particularly members of Calliphoridae and Muscidae [2,14,129,130], while flesh flies (Sarcophagidae) are often delayed colonizers of carcasses [6]. 

Treatment-related differences were detected in selected families: the Muscidae, Ephydridae, and Sarcophagidae; and species: the sarcophagid *Sarcophaga argyrostoma* and ephydrid *Actocetor indicus*. Therefore, the response to envenomization was not expressed as a simple increase or decrease in total fly abundance. The treatment signal was concentrated in particular taxa and at particular postmortem days, rather than being expressed as a broad shift in the pooled fly catch. This distinction is important because carcass-associated flies differ in arrival time, resource use, and association with decomposition stages [2,6,14,129,130]. 

Several considerations temper the biological interpretation of the reported taxon-specific patterns: (a) the affected taxa were substantially less abundant than the dominant ones, and their abundances were characterized by high zero-inflation and greater variability; (b) the treatment-related effects were confined specifically to days 3–6, rather than persisting across the decomposition period; (c) these patterns did not show consistent directional responses across related taxa; for example, Sarcophagidae showed lower abundance on snake-envenomized carcasses, whereas Muscidae showed higher abundance on scorpion-envenomized carcasses, which inconsistency complicates the inference of a generalized venom effect and the increase or decline of some taxa at different times while the total number of flies remained similar among treatments; and (d) these detected differences in selected taxa may reflect treatment-related variation, but they could equally represent stochastic variation within the naturally dynamic process of carcass colonization. Therefore, these observations require independent replication before they can be interpreted as evidence of biologically meaningful venom effects on adult Diptera.

### 4.2. Family-Level Responses to Envenomization

The differential responses among families likely reflect their distinct resource requirements and feeding strategies. The dominant families, Calliphoridae and Muscidae, are typically early colonizers that exploit fresh carcass resources [6,128,130]. Their attraction abundance was primarily driven by the postmortem period, when fresh tissues are available, rather than venom-altered cues. Although Calliphoridae are generally the first dominant early colonizers of carcasses [2,130], Muscidae slightly exceeded them in the present study, primarily in scorpion-envenomized carcasses. This may reflect taxon-specific responses to envenomation rather than a generalized shift in fly succession [42,130]. Several species of Muscidae can use carcasses across different decomposition stages [2,14,130]. Their response in the present study indicates that envenomization may affect some carcass-associated families without producing a detectable change in total fly abundance. Further, Calliphoridae are broadly attracted to sulfur-containing compounds such as dimethyl disulfide and dimethyl trisulfide, which are released during protein degradation [34,35]. While Muscidae shows broader attraction to a range of volatile compounds, including ammonia, carboxylic acids, and alcohols. Calliphoridae showed no treatment effect, whereas Muscidae showed a modest overall treatment effect in addition to a strong postmortem-day effect. The abundance of Calliphoridae was strongly associated with postmortem day, but treatment did not improve model fit. This agrees with the known early association of blow flies with vertebrate remains [2,6,129]. In the present study, the calliphorid pattern was therefore mainly temporal rather than treatment-driven. This distinction is important because Calliphoridae often dominate early carcass colonization, but their abundance may still be governed primarily by decomposition time under some experimental conditions [2,6]. 

Sarcophagidae are often facultative viviparous colonizers that may respond to different cues, including those associated with early tissue degradation [6,131]. The reduced abundance of Sarcophagidae on snake-envenomized carcasses may reflect venom-induced alterations in tissue chemistry that affected the suitability of these carcasses for larviposition. The survival of deposited sarcophagid larvae depends on the immediate availability of suitable tissue; therefore, they may be more sensitive to early tissue changes than ovipositing Calliphoridae, which lay eggs that can withstand greater environmental variability [2,6]. 

Ephydrid flies are often associated with moist substrates, semiliquid decomposing materials, and microbial biofilms [2,132]. Their higher abundance on control carcasses suggests that envenomization may have altered moisture conditions or microbial development in ways that reduced habitat suitability for this family. Scorpion and snake venoms may affect tissue hydration through their effects on vascular permeability and fluid balance [133,134], potentially creating microhabitat conditions that differ between envenomized and control carcasses. Both Ephydridae and Sarcophagidae showed treatment-related temporal variation, but their total abundances were much lower than those of Muscidae and Calliphoridae. The taxon-specific responses in Sarcophagidae and Ephydridae may reflect their sensitivity to more nuanced changes in VOC profiles that do not affect the dominant families. However, we emphasize that this mechanistic interpretation is speculative, as we did not directly measure VOCs or tissue biochemistry. The patterns observed are consistent with these mechanisms but do not provide direct evidence for them. Future studies combining insect sampling with VOC analysis, microbial profiling, and venom residue assessment are needed to test these mechanistic hypotheses.

Phoridae, Ulidiidae, and Drosophilidae were not included in abundance-based GLMM interpretation because of low counts and many zero observations. This decision reduces the risk of unstable estimates and over interpretation. In carcass studies, rare or incidental taxa may still be biologically informative, but their statistical treatment should reflect their limited abundance and occurrence frequency [2,6]. In the present study, these families were therefore retained descriptively rather than used for inferential abundance modeling. 

### 4.3. Species-Level Responses and Forensic Relevance

A central finding of this study is the variation between the dominant species, *Chrysomya albiceps*, *Musca domestica*, and *Musca sorbens*, which showed little or no treatment response, and the less abundant species, *Sarcophaga argyrostoma* and *Actocetor indicus*, which showed detectable treatment-related patterns. *C. albiceps* showed an abundance pattern more closely linked to decomposition time than to the venom treatment, which is consistent with the role of blow flies as early and abundant colonizers of exposed vertebrate remains [2,6,129]. Muscid flies are common members of carcass-associated assemblages, but their abundance and timing can vary according to carcass condition, local habitat, season, and decomposition stage [2,14,130]. The two muscid species, *M. domestica* and *M. sorbens*, showed strong temporal patterns; *M. domestica* showed a treatment effect, while. *Musca sorbens* showed only a marginal treatment effect. These results suggest that muscid flies’ responses should be interpreted at the species level rather than only at the family level. 

Ephydrid and sarcophagid flies, which represented a small fraction of the total assemblage, showed the strongest treatment-related variations. Sarcophagid flies are often recorded after the earliest blow fly activity, although their timing can vary among environments and carcass conditions [2,6,14]. *S. argyrostoma* showed the clearest treatment-related species response. Predicted abundance was lower on snake-envenomized carcasses than on control or scorpion-envenomized carcasses during the middle postmortem period. The present result indicates that *S. argyrostoma* may respond differently from the dominant calliphorid and muscid species under envenomized carcass conditions. Ephydrid flies are not usually the primary forensic indicators on fresh carcasses, but they may occur during later or more advanced decomposition phases, especially when carcass fluids, moist substrates, or altered microhabitats are present [2,6]. *A. indicus* also showed treatment-related temporal variation. However, its abundance was lower than that of the dominant muscid and calliphorid species, and zero observations were frequent. In the present study, *A. indicus* contributed useful species-level information, but its lower abundance limits strong forensic interpretation. These data, on hand, raise an important interpretive question: do effects restricted to minor assemblage components constitute evidence that envenomation influences the adult fly community? On the other hand, these data do not support a community-wide effect but, instead, suggest that envenomation may alter conditions in ways that affect only certain taxa, while the dominant families remain largely unaffected. Consequently, this observation should be interpreted cautiously, as the statistical power for detecting effects in rare taxa is inherently limited.

Rare species were retained descriptively rather than interpreted through abundance-based GLMMs. This included *Chrysomya marginalis*, *Megaselia scalaris*, *Atherigona orientalis*, *Brachydeutera longipes*, *Physiphora smaragdina*, *Discomyza incurva*, *Blaesoxipha rufipes*, and *Leucophenga* sp. In forensic entomology, rare taxa can support ecological interpretation, but not PMI-related inference, which relies mainly on taxa with consistent occurrence, clear temporal patterns, and reliable identification [1,2,6,8,9]. Not all rare taxa recorded here carry the same forensic weight. Species collected as singletons or in very small numbers are better treated as secondary, opportunistic, or incidental records. This applies particularly to *Blaesoxipha rufipes* and *Leucophenga* sp. The single *B*. *rufipes* does not support a regular carcass association, especially because this species is known mainly as a parasitoid of orthopterans [135]. *Leucophenga* sp. was also represented by only three individuals from control carcasses at the dry stage, given the usual association with fungus-rich substrates [136], and thus its occurrence is best treated as incidental. Ulidiids were collected only sporadically from scorpion-envenomized carcasses and may reflect late-stage decomposition substrates rather than a stable treatment response [132]. The phorid fly *Megaselia scalaris* can occur on carcasses as facultative colonizers, so their presence on envenomized carcasses may reflect altered carcass condition instead of attraction to a specific venom treatment [137]. The drosophilid flies were confined to control carcasses only in this experiment and thus add useful faunistic information but not PMI-related interpretation, which is centered on taxa with repeated occurrence, clear temporal structure, and secure identification [2,6,7,8,131]. 

### 4.4. Possible Mechanisms Underlying Taxon-Specific Responses

The treatment-related differences observed at family and species levels may reflect changes in the postmortem cues available to carcass-associated flies. Carcass attraction is strongly influenced by volatile compounds released during tissue breakdown and microbial activity [34,35]. These cues change during decomposition and can affect the timing and intensity of insect arrival [15,18,34]. One possible explanation for the taxon-specific responses observed here is that envenomization may have influenced postmortem cues associated with tissue breakdown and microbial activity. Carcass-associated flies use decomposition-related volatile cues to locate remains, and these cues change during tissue degradation and microbial succession [15,18,34,35]. 

Snake and scorpion venoms differ in their biochemical composition and physiological effects. *W. aegyptia* snake venom is rich in proteolytic and phospholipolytic enzymes that accelerate tissue degradation and may enhance VOCs release [34,105,133]. In contrast, *A. crassicauda* scorpion venom contains neurotoxic peptides, antimicrobial peptides, and other bioactive compounds [28,34,106,134,138]. These venom compounds may alter microbial activity, delay putrefaction, and consequently modify VOCs emission and fly colonization. In addition, these venom differences could contribute to variation in carcass condition after death. Because VOCs, carcass microbiota, tissue chemistry, and venom residues were not analyzed in this study, the link between envenomization and odor-mediated fly attraction remains inferential. We emphasize that this mechanistic interpretation is speculative, as we did not directly measure VOCs or tissue biochemistry. At this stage, volatile cues should be treated as a working explanation, not as a demonstrated pathway. Future studies combining insect sampling with VOC analysis, microbial profiling, and venom residue assessment are needed to test these mechanistic hypotheses.

Fly responses may also be linked to differences in resource use among taxa. Blow flies, muscid flies, flesh flies, and ephydrid flies differ in their colonization patterns and use of carcass resources. Therefore, carcass condition may affect each family differently [2,6,14,22,23,25,114,129,137]. Consequently, a carcass condition that has little effect on one family may affect another family more effectively. This may explain why Calliphoridae was mainly associated with postmortem day, whereas Muscidae, Ephydridae, and Sarcophagidae showed treatment-related patterns.

### 4.5. Forensic Implications of Taxon-Specific Fly Responses

The forensic implication of these results is not that snake or scorpion envenomization currently provides a direct PMI estimation factor, as they are based on adult-fly abundance, not immature developmental age, which remains central to PMI estimation [2,6,7,8,10,130,139]. Rather, the important point is that adult-fly evidence from suspected envenomation cases should not be interpreted from pooled abundance alone. In this experiment, pooled fly abundance remained similar among treatments, whereas selected taxa differed among treatments during particular postmortem windows. Therefore, a suspected venom-related effect may be missed if Diptera are treated as a single group. Family- and species-level identification, combined with postmortem timing, provides a more informative basis for evaluating whether envenomization may have influenced fly activity on a carcass.

The family- and species-level patterns observed here are also relevant to entomotoxicology. Similar effects have also been reported following exposure to drugs and chemical poisons, further supporting that xenobiotics can alter carcass–insect colonization, development, and successional patterns [31,32,33]. Previous studies have shown that drugs, toxins, and venoms may affect decomposition, insect attraction, larval development, or the interpretation of insect evidence [23,24,27,28,29,31,33,38,39,128,132,138,139]. However, these effects are not expected to be identical across taxa. In the present study, treatment-related effects were more evident at the family level for Muscidae, Ephydridae, and Sarcophagidae; and at the species level for the sarcophagid *Sarcophaga argyrostoma* and the ephydrid *Actocetor indicus*. In contrast, the family Calliphoridae and the species *Chrysomya albiceps* were mainly structured by postmortem day.

The forensic value of these findings remains preliminary. The results do not show that snake or scorpion envenomization changed total fly attraction at the whole-assemblage level. They show that some taxa differed in abundance across postmortem days and treatments. If envenomization affects insect evidence, the effect is more likely to appear as a shift in which taxa occur and when they peak, not as a simple rise or fall in the total fly catch. This distinction is important when insect evidence is interpreted from suspected poisoning or envenomation cases. Entomotoxicological evidence can support forensic interpretation, but its value depends on the taxon involved, the biological endpoint examined, and the environmental context of the case [27,28,33]. In the present study, the GLMM approach separated temporal effects from treatment effects. This was important because postmortem day was the strongest driver of abundance, while treatment effects were detected mainly after family- or species-level resolution was applied. 

We suggest three potential forensic applications that may be valuable once validated in future broader studies. The first is that the observed taxon-specific temporal patterns could assist in differentiating between envenomation-related deaths and other causes of death in forensic casework. Two pieces of supportive evidence from the data are (a) the consistent differences in *Sarcophaga argyrostoma* abundance between snake-envenomized and control carcasses could serve as a potential indicator of snake envenomation, and (b) the elevated *Actocetor indicus* abundance on control carcasses during the mid-decomposition period might help in distinguishing the normal death from the envenomation-related death. The second is the utilization of comprehensive morphological and molecular identifications to the species level, particularly in suspected envenomation cases. The third is that our data contribute to enriching the insect succession database for the Riyadh region, which is essential for investigation in forensic casework, and provides valuable ecological context regarding treatment-related variation in adult fly activity in this region. However, we emphasize that these potential applications are preliminary and require substantial validation before they can be implemented in operational forensic practice. Thus, additional studies across seasons, carcass models, geographic settings, and decomposition environments are needed before these patterns can be translated into operational forensic guidance [2,6,14,19,20,22,24,25,114,129].

### 4.6. Limitations of the Present Design

This study was carried out under a controlled field design, and that design also narrows the range over which the results can be applied. All carcasses were exposed during the summer at one semi-natural site in Riyadh city. Local weather, shade, and the activity of the surrounding fly population probably contributed to the observed use of carcasses by Diptera [7,10,13,14,129]. The patterns recorded here should be taken as findings from a single summer experiment at one locality, rather than a general rule for other seasons, habitats, or carcass conditions. We would expect that the results may differ under cooler, more humid, or more variable conditions. Thus, future studies should be conducted across multiple seasons to assess the influence of different meteorological conditions on envenomization-related insect succession patterns.

Rabbits provided a practical and standardized carcass model, as in several forensic studies from Saudi Arabia [14,24,36,94,95,96,140]. Nevertheless, rabbit carcasses differ from larger animal bodies in mass, surface area, skin structure, and heat retention [2,6,91,93]. Such biological differences may alter the pace of tissue breakdown and the period during which the carcass remains suitable for insect colonization. Hence, the results should not be transferred directly to human remains or to larger animal models. 

The analysis was also restricted to adult flies. Adult counts describe attraction and daily occurrence, but they do not provide developmental data. In forensic casework, PMI estimation usually depends more directly on eggs, larvae, pupae, and their developmental age [1,2,6,7,8,10]. The present data are therefore most useful for describing adult fly activity on the carcasses, not for estimating PMI directly. 

The dominant species showed no response, while the responses that were detected occurred in species of lesser abundance. We cannot conclude from these data that envenomation produces a generalized effect on adult fly succession. Rather, the data suggest that some taxa may be more sensitive to altered carcass conditions, while the principal colonizers are robust to venom exposure.

Rare taxa were also treated conservatively, as they occurred in low numbers and produced many zero records. They were retained in the descriptive tables but excluded from abundance-based GLMM interpretation. This approach reduced the risk of unstable estimates while keeping these records available for the faunistic and ecological context. Repeated sampling would be needed before any rare taxon could be linked reliably to envenomized carcasses. 

The mechanism behind the taxon-specific responses remains unresolved. We did not analyze VOCs, microbial profiles, venom residues, or carcass tissue chemistry. As a result, the data cannot separate the possible roles of volatile release, microbial activity, venom persistence, and other postmortem processes. Future experiments should combine insect sampling with VOCs analysis, microbial profiling, and venom-residue assessment.

A further limitation, which deserves particular emphasis, concerns the choice of biological endpoint in this study. We measured adult fly abundance, which has limited precedent as an endpoint in entomotoxicology. The entomotoxicological literature cited in this study has focused mainly on the effects of xenobiotics on the development of immature stages [31,33,128,139], a developmental endpoint of direct forensic significance in PMI estimation. The effect of envenomization on immature stages remains unknown. Future studies should prioritize larval development experiments to determine whether venom exposure affects growth rates, survival, or morphology, as these endpoints have clearer forensic relevance than adult abundance. Until such studies are conducted, the forensic significance of the patterns reported here remains unestablished.

## 5. Conclusions

In suspected envenomation cases, family- and species-level identification may be more informative than pooled adult-fly abundance alone. From a forensic perspective, this study revealed treatment-related, taxon-specificshifts in the succession of carcass flies. Snake and scorpion envenomization did not produce a significant change in the pooled adult-fly catch after nonlinear temporal change and repeated carcass observations were included in the models. The treatment signal appeared mainly at finer taxonomic levels. Muscidae, Ephydridae, and Sarcophagidae showed treatment-related patterns, and the clearest species-level responses were detected in both the sarcophagid, *Sarcophaga argyrostoma*, and the ephydrid, *Actocetor indicus*. Thus, the main contribution of this study is that envenomization effects on adult Diptera, when present, may be taxon-specific and time-dependent rather than assemblage-wide. This reframes the forensic relevance of envenomization: the effect should not be expected to appear as a simple increase or decrease across all adult flies but as taxon-specific variation that may be hidden when the assemblage is pooled. These data do not support a community-wide effect but, instead, suggest envenomation-related alterations in carcass conditions that affect only certain taxa, while the dominant families remain largely unaffected. Consequently, this pattern should be interpreted cautiously and treated as preliminary; it requires validation using immature stages, larger carcass models, different seasons, VOCs data, microbial profiling, and venom-residue analysis before it can be translated into operational PMI guidance. Therefore, we interpret these findings as exploratory and hypothesis-generating rather than as evidence of a broadly applicable forensic pattern.

## Figures and Tables

**Table 1:** Meteorological parameters over the experimental period.

Days PM	Atmospheric Parameters						On-Site Weather Parameters			
	Max.T	Min.T	MT	RH	WT	WS	AT	WT	WS	RH
1	40.0	28.0	34 ± 8.5	5.0	32.0	13.0	29.8	29.0	0.0	32.0
2	43.0	29.0	36 ± 9.9	7.0	31.5	11.0	32.9	33.3	0.0	30.0
3	41.0	30.0	35.5 ± 7.8	6.0	30.2	15.0	38.0	30.0	3.8	30.0
4	42.0	29.0	35.5 ± 9.2	6.0	33.8	13.0	40.0	34.0	0.0	36.0
5	43.0	29.0	36 ± 9.9	7.0	30.0	13.0	29.2	29.7	0.0	30.0
6	43.0	31.0	37 ± 8.5	6.0	30.0	7.4	28.0	28.5	0.0	30.0
7	43.0	39.0	41 ± 2.8	7.0	32.0	5.0	32.0	31.6	0.0	30.0
8	41.0	28.0	34.5 ± 9.2	6.0	30.0	6.0	37.4	34.0	0.0	35.0
9	40.0	23.0	31.5 ± 12.0	12.7	33.9	31.0	38.5	33.0	14.0	30.0
10	36.0	28.0	32 ± 5.6	10.0	34.4	22.2	32.0	30.5	18.0	30.0
11	39.0	28.0	33.5 ± 7.8	13.0	34.5	8.0	36.5	31.5	0.0	36.0
Average	41	29.27	35.14	7.79	32.0	13.1	34.0	31.4	3.2	31.7

PM: postmortem, Max.T: maximum temperature, Min.T: minimum temperature, MT: the mean of maximum and minimum temperatures (*n* = 11), RH: relative humidity, WT: wind temperature, WS: wind speed, and. AT: ambient temperature.

**Table 2:** Summary of negative binomial GLMM results for total and family-level fly abundance.

Response Variable	Selected Model	Tested Effect	χ^2^	df	*p*-Value	Interpretation
Total fly abundance	Treatment + nonlinear day	Treatment	1.26	2	0.533	Not significant
Total fly abundance	Treatment + nonlinear day	Nonlinear day	103.10	3	<0.001	Significant
Total fly abundance	Full interaction model	Treatment × nonlinear day	11.32	6	0.079	Not significant
Muscidae	Treatment + nonlinear day	Treatment	7.10	2	0.029	Significant
Muscidae	Treatment + nonlinear day	Nonlinear day	253.04	3	<0.001	Significant
Muscidae	Full interaction model	Treatment × nonlinear day	7.06	6	0.315	Not significant
Calliphoridae	Nonlinear day only	Treatment	0.46	2	0.795	Not significant
Calliphoridae	Nonlinear day only	Nonlinear day	36.00	3	<0.001	Significant
Calliphoridae	Full interaction model	Treatment × nonlinear day	10.73	6	0.097	Not significant
Ephydridae	Treatment × nonlinear day	Treatment-related effect	43.20	8	<0.001	Significant
Ephydridae	Treatment × nonlinear day	Nonlinear day-related effect	154.04	9	<0.001	Significant
Ephydridae	Treatment × nonlinear day	Treatment × nonlinear day	30.08	6	<0.001	Significant
Sarcophagidae	Treatment × nonlinear day	Treatment-related effect	20.14	8	0.010	Significant
Sarcophagidae	Treatment × nonlinear day	Nonlinear day-related effect	130.85	9	<0.001	Significant

**Table 3:** Significant Tukey-adjusted pairwise contrasts from final GLMMs.

Response Variable	Day	Contrast	Ratio	*p*-Value	Direction
Muscidae	Overall	Scorpion/Snake	1.82	0.032	Scorpion > Snake
Ephydridae	4	Control/Scorpion	6.75	0.005	Control > Scorpion
Ephydridae	5	Control/Scorpion	15.23	<0.001	Control > Scorpion
Ephydridae	5	Control/Snake	6.01	<0.001	Control > Snake
Ephydridae	6	Control/Scorpion	20.51	0.016	Control > Scorpion
Ephydridae	6	Control/Snake	9.78	<0.001	Control > Snake
Sarcophagidae	3	Control/Snake	3.25	0.014	Control > Snake
Sarcophagidae	3	Scorpion/Snake	2.96	0.033	Scorpion > Snake
Sarcophagidae	4	Control/Snake	3.72	0.017	Control > Snake
Sarcophagidae	4	Scorpion/Snake	4.34	0.006	Scorpion > Snake
Sarcophagidae	5	Scorpion/Snake	4.01	0.006	Scorpion > Snake

**Table 4:** Differential attraction of flies’ families and species during the decomposition process post-envenomization with snake and scorpion venoms.

Families (Total Number)	Species (Total Number)	Maximum Count/Carcass-Day (Mean)	Zero Records (%)	Trt	Control				*Walterinnesia aegyptia*				*Androctonus crassicauda*			
				DS	Fr	Bl	De	Dr	Fr	Bl	De	Dr	Fr	Bl	De	Dr
				HPM *	0–31	31–45	45–93	93→	0–21	21–31	31–93	93→	0–21	21–45	45–117	117→
Muscidae (2813)	*M. sorbens* (1485)	9.00 (91)	53.3		●	●	●	●	●	●	●	●	●	●	●	●
	*M. domestica* (1315)	7.97 (92)	59.4		●	●	●	●	●	●	●	●	●	●	●	●
	*A. orientalis* (13)	0.08 (1)	92.1		●	●	●	○	○	○	○	○	●	●	●	○
Calliphoridae (2389)	*C. albiceps* (2356)	14.28 (100)	29.7		●	●	●	●	●	●	●	●	●	●	●	●
	*C. marginalis* (33)	0.20 (3)	89.1		○	●	○	○	●	●	●	○	○	●	○	○
Sarcophagidae (135)	*S*. *argyrostoma* (134)	0.81 (6)	56.4		●	●	●	●	●	●	●	●	●	●	●	●
	*B. rufipes* (1)	0.01 (1)	99.4		○	●	○	○	○	○	○	○	○	○	○	○
Ephydridae (126)	*A. indicus* (110)	0.67 (8)	66.1		●	●	●	●	●	●	●	●	●	●	●	●
	*B. longipes* (12)	0.07 (1)	92.7		●	○	●	●	○	●	●	●	○	○	○	○
	*D. incurva* (4)	0.02 (1)	97.6		○	○	○	○	○	○	●	○	○	●	●	○
Phoridae (19)	*M. scalaris* (19)	0.12 (1)	88.5		○	○	○	○	●	○	●	●	○	○	●	○
Ulidiidae (12)	*P. smaragdina* (12)	0.07 (4)	94.5		○	○	●	○	○	○	●	○	○	●	○	●
Drosophilidae (3)	*Leucophenga* sp. (3)	0.02 (3)	99.4		○	○	○	●	○	○	○	○	○	○	○	○

Trt: treatments; Ds: decomposition stages; Fr: fresh; Bl: bloating; De: decayed; Dr: dry; HPM: hours postmortem; *: the duration (start-to-end hours) of each decomposition stage. The arrow (**→**) indicates extended duration. Filled dot (●): indicates recorded flies, while an open dot (○) indicates no record. Shaded rows represent predominant species (attracted to all experimental carcasses). Values are based on 165 carcass-day observations per species.

**Table 5:** Summary of the negative binomial GLMM analysis of the fly species-level differential succession post-envenomization with snake and scorpion venoms.

Species	Selected Model	Effect Tested	χ^2^	df	*p*-Value	Interpretation
*C. albiceps*	NL day only	Treatment-NL day	10.59	6	0.102	Not significant
*C. albiceps*	NL day only	Treatment	0.37	2	0.829	Not significant
*C. albiceps*	NL day only	NL day	37.40	3	<0.001	Significant
*M. domestica*	Treatment + NL day	Treatment-NL day	10.21	6	0.116	Not significant
*M. domestica*	Treatment + NL day	Treatment	6.66	2	0.036	Significant
*M. domestica*	Treatment + NL day	NL day	210.42	3	<0.001	Significant
*M. sorbens*	Treatment + NL day	Treatment-NL day	8.45	6	0.207	Not significant
*M. sorbens*	Treatment + NL day	Treatment	6.01	2	0.050	Marginal
*M. sorbens*	Treatment + NL day	NL day	232.05	3	<0.001	Significant
*S. argyrostoma*	Treatment × NL day	Treatment-NL day	18.50	6	0.005	Significant
*S. argyrostoma*	Treatment × NL day	NL day-related effect	127.73	9	<0.001	Significant
*A. indicus*	Treatment × NL day	Treatment-related effect	33.52	8	<0.001	Significant, cautious
*A. indicus*	Treatment × NL day	NL day-related effect	139.36	9	<0.001	Significant, cautious

GLMM: general linear mixed models; NL: nonlinear.

**Table 6:** Significant Tukey-adjusted species-level pairwise contrasts.

Species	Day	Contrast	Ratio	*p*-Value	Direction
*S. argyrostoma*	3	Control/Snake	3.24	0.014	Control > Snake
*S. argyrostoma*	3	Scorpion/Snake	2.95	0.034	Scorpion > Snake
*S. argyrostoma*	4	Control/Snake	3.66	0.019	Control > Snake
*S. argyrostoma*	4	Scorpion/Snake	4.27	0.006	Scorpion > Snake
*S. argyrostoma*	5	Scorpion/Snake	3.95	0.007	Scorpion > Snake
*A. indicus*	4	Control/Scorpion	6.70	0.009	Control > Scorpion
*A. indicus*	4	Control/Snake	3.30	0.046	Control > Snake
*A. indicus*	5	Control/Scorpion	11.67	0.004	Control > Scorpion
*A. indicus*	5	Control/Snake	6.96	0.001	Control > Snake
*A. indicus*	6	Control/Snake	9.36	0.005	Control > Snake

## Data Availability

The original contributions presented in this study are included in the article. Further inquiries can be directed to the corresponding author.

## Supplementary Materials

The following supporting information can be downloaded at: https://www.mdpi.com/article/10.3390/insects17080863/s1. Figure S1: A plate shows A: view of the botanical garden used as experimental site, B: a fresh-stage rabbit carcass in its protective metal cage along with pitfall traps, C: the desert black snake, *Walterinnesia aegyptia*, D: the fat-tail scorpion, *Androctonus crassicauda* and E: the ear intravenous injection of rabbits; Table S1: Descriptive abundance summary of dipteran families.
