# Supplementary material for: Envenomization with Snake Versus Scorpion Venom: Postmortem Repercussions on the Successional Dynamics of Carcass-Attracted Flies in a Forensic Context
Source: Insects. 2026 Aug 19;17(8):863. doi: 10.3390/insects17080863 (PMC13513510; doi:10.3390/insects17080863)
Supplement: Supplementary file 1 [file insects-17-00863-s001.zip › insects-4349246-supplementary.pdf]

Supplementary File

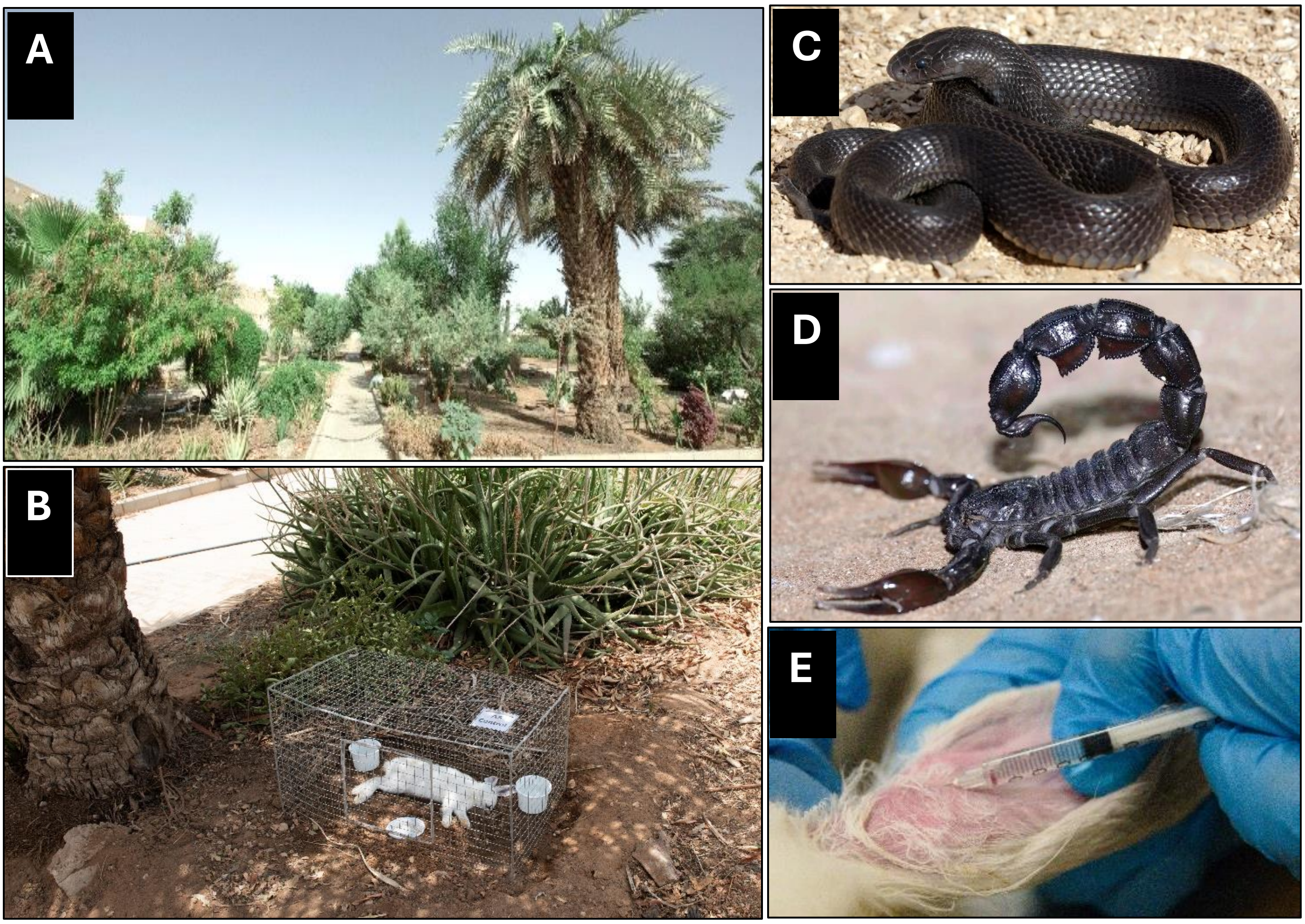

Supplementary Figure S1. A plate shows A: view of the botanical garden used as experimental site, B: a fresh-stage rabbit carcass in its protective metal cage along with pitfall traps, C: the desert black snake, *Walterinnesia aegyptia*, D: the fat-tail scorpion, *Androctonus crassicauda* and E: the ear intravenous injection of rabbits.

Supplementary Table S1. Descriptive abundance summary of dipteran families

Family	Numbers	Mean number per carcass-day	Zero records (%)	Maximum number per carcass-day
Muscidae	2,813	17.05	49.7	184
Calliphoridae	2,389	14.48	29.7	90
Sarcophagidae	135	0.82	56.4	6
Ephydridae	126	0.76	64.2	8
Phoridae	19	0.12	88.5	1
Ulidiidae	12	0.07	94.5	4
Drosophilidae	3	0.02	99.4	3
**Total number**	5,497			

Values are based on 165 carcass-day observations per family. Muscidae, Calliphoridae, Sarcophagidae, and Ephydridae were evaluated using negative binomial generalized linear mixed models. Phoridae and Ulidiidae were summarized descriptively because of their very low abundance and high proportions of zero counts.
